# Chitosan-Based Functional Materials for Skin Wound Repair: Mechanisms and Applications

**DOI:** 10.3389/fbioe.2021.650598

**Published:** 2021-02-18

**Authors:** Peipei Feng, Yang Luo, Chunhai Ke, Haofeng Qiu, Wei Wang, Yabin Zhu, Ruixia Hou, Long Xu, Songze Wu

**Affiliations:** ^1^School of Medicine, Ningbo University, Ningbo, China; ^2^Lihuili Hospital, Affiliated Hospital of Ningbo University, Ningbo, China; ^3^School of Materials Science and Chemical Engineering, Ningbo University, Ningbo, China; ^4^Ningbo Baoting Biotechnology Co., Ltd., Ningbo, China

**Keywords:** chitosan, functional materials, hydrogels, wound repair, mechanisms

## Abstract

Skin wounds not only cause physical pain for patients but also are an economic burden for society. It is necessary to seek out an efficient approach to promote skin repair. Hydrogels are considered effective wound dressings. They possess many unique properties like biocompatibility, biodegradability, high water uptake and retention etc., so that they are promising candidate materials for wound healing. Chitosan is a polymeric biomaterial obtained by the deacetylation of chitin. With the properties of easy acquisition, antibacterial and hemostatic activity, and the ability to promote skin regeneration, hydrogel-like functional wound dressings (represented by chitosan and its derivatives) have received extensive attentions for their effectiveness and mechanisms in promoting skin wound repair. In this review, we extensively discussed the mechanisms with which chitosan-based functional materials promote hemostasis, anti-inflammation, proliferation of granulation in wound repair. We also provided the latest information about the applications of such materials in wound treatment. In addition, we summarized the methods to enhance the advantages and maintain the intrinsic nature of chitosan via incorporating other chemical components, active biomolecules and other substances into the hydrogels.

## Introduction

Skin wounds are ubiquitous conditions caused by surgery, scolding, grazing, chronic ulcers, and other trauma. Skin wound healing is a physiological process affected by multiple factors, which can be accelerated by proper wound treatment with effective wound care materials. Ideal wound care materials should be flexible, stable, biodegradable, and widely applicable, with the ability to keep wounds moist, stop bleeding, and adsorb exudate (Abd El-Hack et al., 2020).

With in-depth investigations into the molecular biological mechanisms regarding wound healing and the fast development of tissue engineering biomaterials, a variety of novel biomaterials and hydrogel-like materials have received the most interest attention. Hydrogel is a 3D structural system formed by the crosslinking of various hydrophilic polymeric chains (Lee et al., 2019). With both the viscoelasticity of solids and the fluidity of liquids, hydrogels possess similar structural and functional characteristics to extracellular matrices. The advantages of hydrogels over other typesof wound dressings include: (1) superior hydrophilicity, which enables the hydrogels to adsorb exudate (Khorasani et al., 2018);(2) the ability to keep wounds moist (Zhang et al., 2020); (3) the ability to form a physical barrier against microbial contamination of the wound (Khorasani et al., 2018; Qu et al., 2018); and (4) low adhesion force that prevents repeated damage from dressing changes.

**FIGURE 1:**
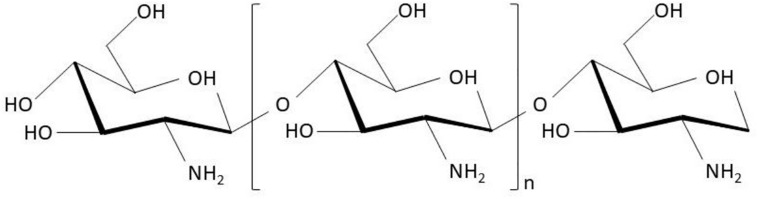
Scheme of chitosan chemical structure [cited from literature (Abd El-Hack et al., 2020)].

**FIGURE 2:**
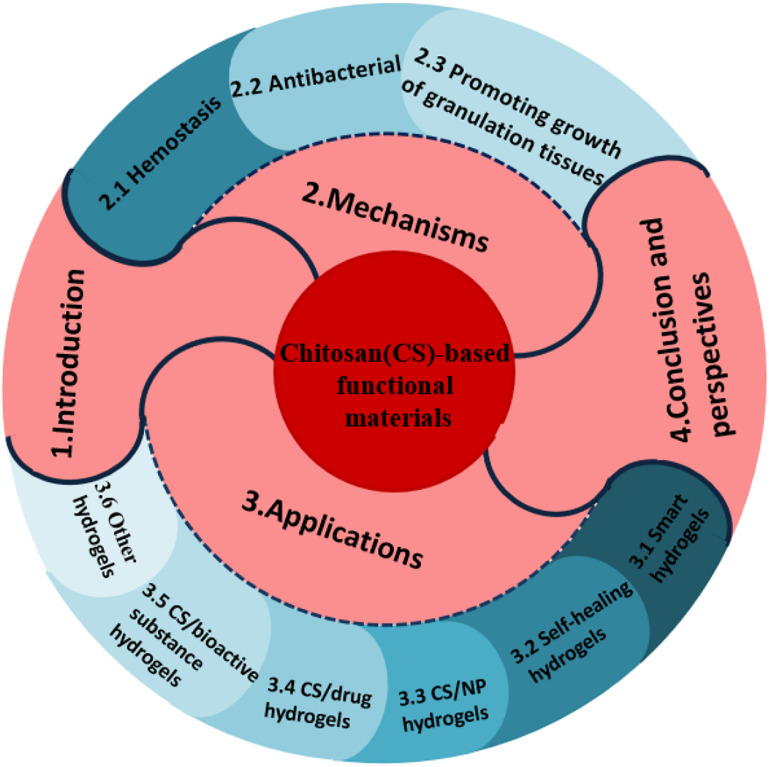
The work flow of the whole article.

Generally, hydrogels can be classified into native hydrogels and synthetic hydrogels. Native hydrogels are prepared using native polymers including chitosan, sodium alginate, collagen, and sodium hyaluronate as ingredients. Among these, chitosan is one of the most frequently studied and attractive hydrogels. Chitosan (also known as (1 → 4)-2-amino-2-deoxy-β-D-glucan), formed by *N*-acetyl-D-glucosamine monomers with β-1,4-glycosidic bonds (Figure 1), can be acquired by the deacetylation of chitin extracted from crustacean shells. Many benefits of chitosan and its derivatives on skin wounds have been reported, including: (1) desirable pharmacological actions like antibacterial (Mohan et al., 2020), anti-inflammatory, hemostatic (Khan and Mujahid, 2019), and skin regenerative behavior (Mohan et al., 2020); (2) superior biocompatibility and biodegradability (Li et al., 2020); (3) good water-absorption and water-retention properties; and (4) amino (-NH_2_) and hydroxyl (-OH) groups on the molecular chains, which allow the grafting of other groups and chemical components for enhancing certain biological functions (Abd El-Hack et al., 2020). This article reviewed the functions and mechanisms of chitosan as a wound care material to inhibit bacterial infections, stop bleeding, and promote the growth of granulation tissues, along with the applications of chitosan as hydrogels for wound treatment. The work flow of this article is summarized as Figure 2.

## Effects and Mechanisms of Chitosan on Skin Wounds

The whole wound healing procedure generally includes four stages which named as hemostasis, inflammation, proliferation, and skin remodeling. In the hemostasis stage, the coagulation system is activated after blood vessels constrict and platelets aggregate. Fibrinogen is transformed into insoluble fibrin that forms clots to stop hemorrhaging. In the inflammation stage, bacteria and necrotic tissue are cleared by inflammatory cells. Epithelial cells proliferate and migrate to form epithelial tissue to cover the wound in the proliferation stage. Granulation tissue fills the tissue gap, but epithelialization does not take place. During the final remodeling stage, fresh epidermis and dermis will regenerate to finish the skin repair procedure.

**FIGURE 3:**
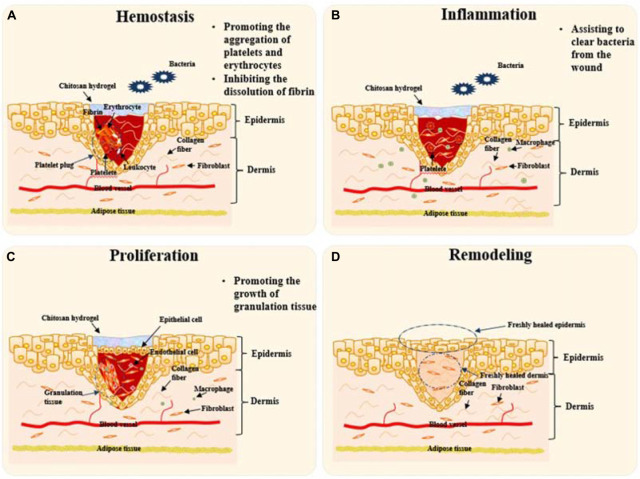
Schematic diagram to illustrate the mechanisms of chitosan-based hydrogels to promote wound healing. Remodeling will take place by skin tissue after the “Proliferation” stage. **(A)** Platelet plugs composed of platelets, leukocytes, insoluble fibrin, and erythrocytes prevent bleeding at the stage of Hemostasis. Chitosan hydrogel can help to stop hemorrhaging via promoting the aggregation of platelets and erythrocytes and inhibiting the dissolution of fibrin. **(B)** At “Inflammation” stage, chitosan hydrogels will assist inflammatory cells like macrophages to clear bacteria and necrotic tissue from the wound. **(C)** Epithelial cells proliferate and migrate to form epithelial tissue to cover the wound at the stage of “Proliferation”. Chitosan hydrogel promotes the growth of granulation tissue towards filling the tissue gap. **(D)** The final stage, remodeling takes place to finish the whole procedure of skin repair. Chitosan-based hydrogels take effects mainly in the first three stages.

Chitosan and its derivatives will play roles mainly in the first three stages during wound healing. Firstly, they help stop hemorrhaging by promoting the aggregation of platelets and erythrocytes and inhibiting the dissolution of fibrin in the hemostasis stage; Secondly, they can assist to clear bacteria from the wound during the inflammation stage; Finally, they accelerate skin proliferation via promoting the growth of granulation tissue, which is called proliferation stage. After then, the wound was healed and the skin was remodeled to finish the healing route. Figure 3 illustrates schematically the mechanisms of chitosan-based hydrogels to promote wound healing at the first three stages. The roles played by chitosan-based materials in these three aspects are discussed in detail as follows.

### Hemostasis

As the first step in wound healing, hemostasis sets the foundation for the subsequent phases. Physiologically, the hemostasis process is composed of four steps. (1) In the vasoconstriction step, the wound triggers reflex vasoconstriction. (2) Platelet aggregation forms platelet plugs. (3) Fibrin clots are formed by the activation of the coagulation system. The coagulation system involves three pathways: extrinsic coagulation, intrinsic coagulation, and common coagulation. The extrinsic coagulation pathway starts with the release of factor III after blood vessels are damaged. The released factor III activates factor VII, which in turn, reacts with factor III and Ca^2+^ to form a compound called activated factor X (AFX). The intrinsic coagulation pathway starts with the activation of factor XII caused by the exposure of collagen fibers after blood vessel damage. Activated factor XII then activates factor XI, which in turn, activates factor IX. Factor X is then activated by the compound formed by Ca^2+^, AF3, AFV, and PF3. The common coagulation pathway utilizes AFX produced in the extrinsic and intrinsic coagulation pathways. AFX reacts with Ca^2+^, PF3, and AFV to form the prothrombin activator, which activates thrombin. Thrombin is capable of transforming fibrinogen into fibrin, which produces a network to agglomerate erythrocytes, leukocytes, and platelets, creating fibrin clots that contribute to the coagulation process. (4) Fibrin clot dissolution occurs after the wound stops bleeding when inactive plasminogen is converted to active plasmin by plasminogen activators. This step dissolves blood clots to prevent vessel blockage.

However, the superior hemostatic effect of chitosan is not related to the classic coagulation system (Leonhardt et al., 2019; Wang et al., 2019), i.e., not the activation of coagulation factors (Khan and Mujahid, 2018). Chitosan promotes platelet adhesion and aggregation, inducing erythrocyte aggregation and inhibiting fibrinolysis.

### Promoting Platelet Adhesion and Aggregation

**FIGURE 4:**
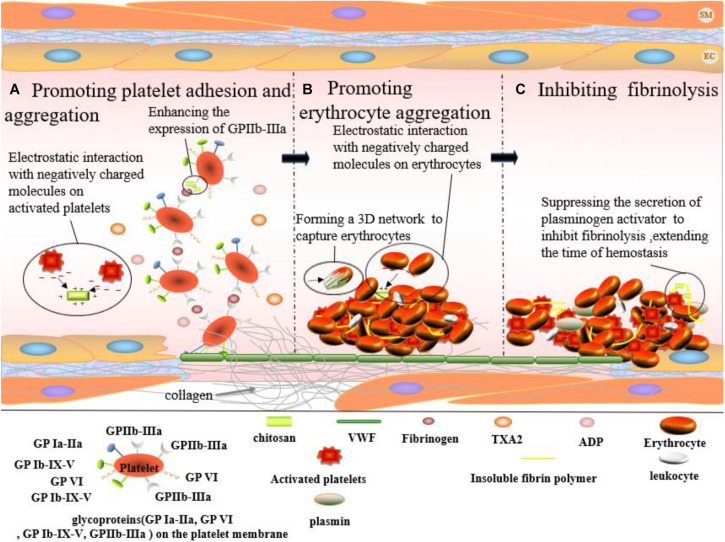
Hemostatic effect of chitosan on skin wound which occurs at the first stage of wound healing. **(A)** Chitosan enhances the expression of GPIIb-IIIa from platelet. And, positively charged chitosan can interact with negatively charged molecules on the activated platelets, promoting platelet aggregation. **(B)** Erythrocytes aggregate via the interaction between positively charged chitosan and negatively charged molecules on erythrocyte surface. And, chitosan accelerates the formation of fibrin clots by forming a 3D network to capture erythrocytes (black arrows point chitosan). **(C)** Chitosan plays a hemostatic role by inhibiting fibrinolysis.

Platelet adhesion and aggregation are crucial steps in hemostasis. This process relies upon glycoprotein (GP) Ia-IIa, GP VI, GP Ib-IX-V, and GP IIb-IIIa present on the platelet membrane, subendothelial collagen, as well as von Willebrand as well as VWF and fibrinogen in plasma factor (VWF) and fibrinogen in plasma (Figure 4A). When endothelial cells are damaged, the underlying collagen is exposed. Spherical VWF binds to the collagen surface and becomes thread-like under blood flow. The allosteric VWF rapidly binds to GP Ib-IX-V, preventing platelets from flowing away from the wounded site. Platelets are retained by binding to GP VI on the surface of collagen. The binding of VWF and GP Ib-IX-V activates the relevant signaling pathways in platelets, in turn, activating GP Ia-IIa and GP IIb-IIIa. Finally, activated GP Ia-IIa and GPIIb-IIIa bind to collagen and VWF, keeping platelets fixed on the collagen surface. The release of ADP and TXA2 from platelets is attributed to the activation of the signaling pathways caused by the binding between VWF and GP Ib-IX-V and between GP VI and collagen. ADP and TXA2 further activate GPIIb-IIIa on the nearby platelet membranes. The bridging between activated GP IIb-IIIa and fibrinogen results in the aggregation of platelets and thus, the formation of platelet plugs. Chitosan is capable of enhancing GPIIb-IIIa expression on platelet membranes (Lord et al., 2011), thereby promoting the adhesion of platelets to the vascular wall and the aggregation of platelets (Jackson et al., 2005). Moreover, positively charged chitosan can also promote platelet aggregation by interacting with the massive quantity of negatively charged substances on the surface of the activated platelets (Wang et al., 2019).

### Promoting Erythrocyte Aggregation

The fibrin network agglomerates erythrocytes, leukocytes, and platelets to create fibrin clots. The surface of erythrocytes is negatively charged due to neuraminic acid residues on their membranes. Fibrin clot formation and erythrocyte aggregation can be promoted by electrostatic interactions between positively charged chitosan and negatively charged groups on the erythrocyte surface (Figure 4B) (Ong et al., 2008). He et al. (2013) prepared a series of chitosan membranes with different protonation degrees by NaOH deacidification. The study reported a positive correlation between the affinity of chitosan for erythrocytes and the degree of protonation. Moreover, chitosan could capture and agglomerate erythrocytes by forming a 3-D network in blood, thereby promoting fibrin clot formation (Wang et al., 2008).

### Inhibiting Fibrinolysis

During fibrinolysis, fibrin is dissolved by plasmin. Fibrin clots disappear and normal blood flow is restored. However, it desirable to inhibit fibrinolysis, prolonging the existence of fibrin clots, and thus, extending hemostasis. Chitosan is capable of inhibiting fibrinolysis (Figure 4C). As early as 1992, the 80% deacetylation chitosan was reported to inhibit the secretion of plasminogen activator by macrophages and, therefore, inhibited fibrinolysis, exhibiting hemostatic effects in rabbit peritoneal trauma (Fukasawa et al., 1992).

### Antibacterial Mechanisms of Chitosan

Wounded skin undergoes a series of complex repairing processes, including hemostasis, coagulation, inflammation, angiogenesis, granulation tissue development, and re-epithelialization. The moist and nutrition-rich environment of the wound provides desirable conditions for bacterial growth. Bacterial infections occur when the host immune system fails to clear all invading bacteria. Therefore, the antibacterial properties of wound dressings need to be seriously considered. Chitosan is widely used in wound treatment due to its superior antibacterial properties (Li et al., 2019), but its antibacterial mechanisms are still unclear. Currently, the acknowledged possible mechanisms include disrupting bacterial cell walls and cell membranes, chelating trace amounts of metallic cations, interacting with intracellular targets, and depositing on bacteria.

### Disrupting the Cell Wall and Cell Membrane of Bacteria

**FIGURE 5:**
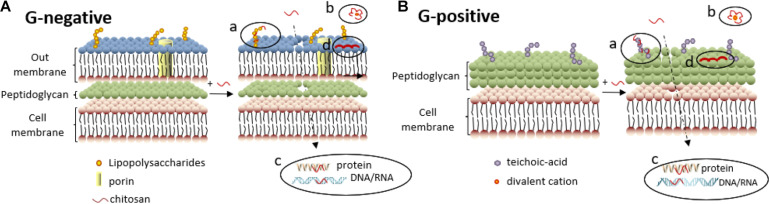
Antibacterial mechanisms of chitosan against Gram-negative **(A)** and Gram-positive bacteria **(B)**. **(a)** Electrostatic interactions between chitosan and lipopolysaccharides (or teichoic -acid) disrupt the cell membrane, enabling chitosan to penetrate further into the cell membrane. **(b)** Divalent cations are chelated by chitosan, decreasing the stability of the outer membrane. **(c)** Chitosan inside the cell can inhibit synthesis of DNA/RNA and protein, further inhibit Gram-negative bacteria proliferation. **(d)** High-molecular-weight chitosan deposition on the surface of Gram-negative bacteria hinders bacterial metabolism.

Bacteria can be classified into Gram-negative bacteria and Gram-positive bacteria according to Gram staining results. The cell wall of Gram-negative bacteria is comprised of an outer membrane and a peptidoglycan layer (Figure 5A). The outer membrane comprises two asymmetric monolayers. The inner layer is solely composed of phospholipids, while the outer layer is composed of phospholipids and lipopolysaccharides. The surface of Gram-negative bacteria is negatively charged owing to the phosphate and pyrophosphate groups of lipopolysaccharides in the outer layer. The cell wall of Gram-positive bacteria is comprised of peptidoglycans and teichoic acids (Figure 5B). The surface of Gram-positive bacteria is negatively charged owing to the carboxyl and phosphate groups of teichoic acids.

When chitosan with high molecular weight is dissolved in acidic aqueous solutions, the NH_2_ groups are protonated to -NH_3_^+^ cations (Bernkop-Schnürch, 2018). The electrostatic interactions between -NH_3_^+^ and lipopolysaccharides on the cell membrane of Gram-negative bacteria as shown in Figure 5A(a) or teichoic acids on Gram-positive bacteria as shown in Figure 5B(a) will lead to the uneven distribution of negative charges on bacteria (Kassem et al., 2019), resulting in an imbalance between cell wall synthesis and dissolution (with a stronger tendency toward dissolution). The bacterial cell membrane is deformed and ruptured under the unsustainable osmotic pressure, leading to cell content leakage and eventually cell lysis.

Cell membrane disruption can be deduced by the leakage of ions such as K^+^, PO_4_^3–^,and macromolecules such as DNA and RNA, which are detected by strong UV absorption at a wavelength of 260 nm (Li et al., 2015). Xing et al. (2009a) detected an adsorption peak at 260 nm after contact between oleoyl chitosan and *Staphylococcus aureus* (*S. aureus*) for 5 min. The results of SEM observation disclosed that chitosan molecules were adhered to the surface of *S. aureus* and *Escherichia coli* (*E. coli*) after 30 min of contact, and cell wall disruption and cell content leakage were observed for both bacterial strains.

Apart from the bactericidal activities against both Gram-negative and Gram-positive bacteria, chitosan also exhibits antifungal properties. The antifungal effectiveness of chitosan is positively correlated with the fluidity of the cytoplasmic membrane, which depends upon the amount of PUFAs (Verlee et al., 2017). The resistance of fungi to the bactericidal effect of chitosan is divided into chitosan-resistant fungi and chitosan-sensitive fungi. The intrinsic fluidity is very low for fungi with relatively small amounts of PUFAs. The binding between chitosan and negatively charged phospholipids cannot substantially affect the fluidity of the cytoplasmic membrane, and therefore, exhibits little antifungal activity by the inability to alter the permeability of the cytoplasmic membrane. Fungi that can resist the antifungal activity of chitosan are called chitosan-resistant fungi. Fungi that cannot effectively resist the antifungal activities of chitosan are called chitosan-sensitive fungi.

### Chelating Metallic Cations

Divalent cations can stabilize the membrane structure of bacteria. Clifton et al. (2015) developed the GNB-OM model to test the stabilizing effect of divalent cations. The results showed that the salt bridges formed by divalent cations and the negatively charged oligosaccharides of lipopolysaccharide are crucial to the structural integrity of the outer cytoplasmic membrane. The negative charges of the lipopolysaccharide molecules are neutralized by hydrogen bonds and cations, forming networks impermeable to macromolecules and hydrophobic molecules. The phosphate groups in the cell wall of Gram-positive bacteria can also utilize divalent cations like Mg^2+^ and Ca^2+^ to maintain the stability of the cytoplasmic membrane.

Chitosan is a type of chelating agent. When the pH value of the system falls below the isoelectric point of chitosan and its derivatives, protonated -NH_3_^+^ in the molecular chains are attracted by negative charges while chitosan and its derivatives chelate the divalent cations on the cell membrane surface [Figures 5A(b),B(b)], leading to an imbalance in the surface potential and a mutual repulsion among the negatively charged molecules, and finally, rupture of the cell membrane.

### Interacting With Intracellular Targets

Chitosan with a molecular weight of no more than 5000 D can penetrate the bacterial cell wall to form complexes with DNA, undermining the function of DNA polymerase and RNA polymerase, and thereby, suppressing the replication and transcription of DNA and RNA [Figures 5A(c),B(c)], which inhibits bacterial proliferation (Farhadihosseinabadi et al., 2019). Xing et al. (2009b) evaluated the binding ability of OCNPs to DNA/RNA by characterizing the mobility of the bacterial genome on agarose gel electrophoresis. It was found that the brightness of the electrophoretic band diminished with increasing OCNP concentrations. The migration of DNA and RNA from *E. coli* was completely suppressed by 1,000 mg/L OCNPs, presumably due to the electrostatic attraction between the positively charged amino groups of the OCNPs and the negatively charged phosphate groups in the nucleic acids of *E. coli*.

Moreover, chitosan with low molecular weight inhibited the protein synthesis of microorganisms [Figures 5A(c),B(c)] (Galván Márquez et al., 2013). Galván Márquez et al. (2013) investigated a collection of approximately 4,600 gene deletion mutants of *Saccharomyces cerevisiae* (*S. cerevisiae*) using a yeast gene deletion array to explore the chemical-genetic interactions between chitosan and *S. cerevisiae*. The results showed that of the 107 mutants most sensitive to chitosan, 31% had gene deletions related to protein synthesis. The electrostatic interactions between the positively charged amino groups from chitosan and the negatively charged carboxyl groups from proteins were responsible for the inhibition of protein synthesis.

### Depositing on Bacterial Surfaces

When dissolved in acidic aqueous solutions, chitosan with high molecular weight can form a dense polymeric layer on the bacterial surface that prevents the intake of nutrients or the excretion of metabolites, leading to metabolic disorders and bacterial death [Figures 5A(d),B(d)]. This flocculation effect was verified by SEM observation, showing vesicle-like structures on the outer membrane of chitosan-treated *E. coli* and *Salmonella typhimurium* (*S. typhimurium*) (Helander et al., 2001).

### Promoting Growth of Granulation Tissues

Tissue regeneration and skin repair start immediately after the skin is wounded. One of the indispensable stages in skin repair is the formation of granulation tissues composed of inflammatory cells, fibroblasts, and new capillaries. Granulation tissue is capable of refilling the wounded area and promoting epidermal regeneration. Research has shown that chitosan can accelerate skin wound repair by promoting the growth of inflammatory cells (represented by macrophages), fibroblasts, and capillaries. For macrophages, chitosan can promote the secretion of cytokines such as transforming growth factor-β (TGF-β), PDGF, and IL-1. TGF-β induces the migration of macrophages to wounded areas, promoting fibroblast proliferation and enhancing collagen secretion. During skin regeneration, PDGF can enhance angiogenesis and stimulate the migration and proliferation of fibroblasts, and promote the synthesis of glycosaminoglycans, proteoglycans, and collagen, all of which are beneficial to the formation of granulation tissue. IL-1 is also known to help wound healing by promoting angiogenesis, fibroblast proliferation, and collagen synthesis. Additionally, chitosan can increase the secretion of IL-8 from fibroblasts, which can accelerate the inflammation process and stimulate angiogenesis. The impact of chitosan on fibroblast proliferation depends upon its molecular weight and deacetylation degree. Chitosan with a high deacetylation degree and low molecular weight has a more pronounced effect to promote fibroblast proliferation (Howling et al., 2001).

## Function and Application of Hydrogel-Like Chitosan-Based Materials in Wound Treatment

Chitosan is extensively used as a functional material for wound treatment due to its hemostatic effect in the early stages and the ability to inhibit microbial growth and accelerate wound healing. Chitosan can be utilized in forms such as membranes, hydrogels, fibers, sponges. Hydrogel-like chitosan has received the most attention because of its advantages over other forms of chitosan, including better flexibility, high water content, ability to adsorb exudate, permeability to oxygen, and proper cooling effect that alleviates pain. Nevertheless, there are certain problems in preparing hydrogels using chitosan alone. For example, chitosan is soluble only in weakly acidic solutions, with relatively weak mechanical strength and deficiencies in certain functions. To meet the requirements in the field of skin wound repair, modifying techniques such as introducing other chemical components are usually applied. The applications of chitosan hydrogels in skin wound repair are summarized in Table 1.

**TABLE 1:** Summary of chitosan-based hydrogels containing functional components.

Type	Composition	Functional component	Applications in wound healing	Advantages	References
Smart hydrogels	CS, tempo-oxidized cellulose nanofiber (TOCNF), and β-glycerophosphate (thermo-sensitive)	β-glycerophosphate molecule and TOCNF nanofiber.	Skin wound	Incorporation of TOCNF improved acute response with more prominent M2 macrophage cells	Nguyen et al., 2018
	CS, hydroxypropyl methylcellulose (HPMC) and glycerol (thermo-sensitive)	CS and HPMC molecule	Skin wound	Low-cytotoxicity, biodegradable and controlled release	Wang et al., 2016
	Umbilical cord-derived mesenchymal stem cell-derived exosomes (hUCMSC-exos) and poloxamer 407 (thermo-sensitive)	Nano-scale hUCMSC-exos	Diabetic chronic wound	Sustainedly hUCMSC-exos release; promoted angiogenesis; accelerated wound healing rate; improved epithelium regeneration	Yang et al., 2020
	CMCS, sodium alginate (SA), photosensitive sinoporphyrin sodium (DVDMS), and poly(lactic-co-glycolic acid)-encapsulated basic fibroblast growth factor (PLGA-bFGF) (light-responsive)	PLGA-bFGF nanospheres	Burned and infected wound	Antibacterial activity against *multidrug-resistant S. aureus*; Rapid wound closure and highly efficient sterilization.	Mai et al., 2020
Self-healing hydrogels	CS, oxidized konjac glucomannan (OKGM), and silver/Ag nanoparticles (AgNPs)	OKGM molecule and Ag NPs	Irregular wound	Antibacterial activity against *E. coli* and *S. aureus*;	Wang et al., 2020
	CMC and dialdehyde-modified cellulose nanocrystal (DACNC)	DACNC nanocrystal	Deep but partial thickness burned wound	Excellent biocompatibility; Suppression of scar formation	Huang et al., 2018
	CS and vanillin (porous 3D hydrogel)	Vanillin molecule	Skin wound	Oxygen and nutrient transportation in wound-repairing	Xu et al., 2018
Nanoparticles (NPs) containing CS hydrogels	OKGM, CMCS, and AgNPs	Ag NPs	Chronic wound	Improved antibacterial activity against *E. coli* and *S. aureus*; excellent biocompatibility	Jiang et al., 2020
	polyvinyl alcohol (PVA), CS and Ag NPs	Ag NPs	Chronic ulcer	Antimicrobial activity on *E. coli* and *S. aureus*; The controlled silver release	Kumar and Kaur, 2020
	Heparinized PVA, CS, and zinc oxide (ZnO) NPs	ZnO NPs	Skin wound	Antimicrobial activity on *E. coli* and *S. aureus*; Protection against dehydration and exudate accumulation	Khorasani et al., 2018
	CS-pluronic and Cu NPs	Cu NPs	Anti-microbia as wound dressing	Antimicrobial activity on *E. coli* and *S. aureus*.	Jayaramudu et al., 2020
Drug containing CS hydrogels	Chitosan-cetyltrimethylammonium bromide -based hydrogel (CCH), mupirocin, and Se NPs	Mupirocin molecule and Se NPs	Diabetic and *Mupirocin-methicillin-resistant Staphylococcus aureus* (MMRSA) infected wound	Improved wound contraction; improved angiogenesis, fibroelastosis, collagenesis, and proliferation of hair follicle and epidermis; Antimicrobial activity on MMRSA	Golmohammadi et al., 2020
	Poloxamer, CS, HA, Vitamin A, D and E	Molecules of Vitamin A, D and E	Burned wound	Antibacterial activity against all strains except for *E. coli*; accelerated skin regeneration; improved antioxidant capacity	Soriano-Ruiz et al., 2020
	Alginate, CS and 10% hesperidin	Hesperidin molecule	Deep wound	No cytotoxicity; improved collagen synthesis; High wound closure percentage	Bagher et al., 2020
Chitosan/bioactive substance hydrogels	Adipose-derived stem cells (ASCs), CS, and gelatin	ASCs	Therapeutic angiogenesis	Promoted fibroblast migration and angiogenesis	Cheng et al., 2017
	Bone marrow-derived mesenchymal stem cells (BMSCs) and hydroxybutyl chitosan-Arg-Gly-Asp (HBC-RGD)	BMSCs	Keloid treatment	Suppression of keloid fibroblast proliferation and nodular collagen fibers	Qu et al., 2019
	Ag^+^, bFGF, and CS	bFGF and Ag^+^	Infected chronic wound	Improved neovascularization; reduced inflammatory response; promoted M2 polarization in wound	Xuan et al., 2020
	EGE, bFGF, and GCH	EGE and bFGF	Skin wound	Promoted fibroblasts proliferation, collagen, re-epithelialization and granulation tissue formation	Yoo et al., 2018
Other CS composite hydrogels	CS, PVA, and *S*-nitroso-*N*-acetyl-DL-penicillamine (SNAP)	SNAP molecule	Diabetic or burned wound	Enhanced angiogenesis and wound closure on diabetic chronic burned wounds	Zahid et al., 2019a
	CS, PVA, and SNAP	SNAP molecule	Diabetic ulcer or burned wound	Increased proliferation and speeded migration of 3T3 fibroblasts	Zahid et al., 2019b
	Lignin, CS, and PVA	Molecules of CS and lignin	High efficient wound care	Increased mechanical strength; accelerated wound healing due to antioxidant activity	Zhang et al., 2019

### Smart Chitosan-Based Hydrogels

Smart chitosan hydrogels are responsive to external stimuli and have become a focal point of research in the past few years. Generally, smart chitosan hydrogels are categorized into thermosensitive hydrogels, photosensitive hydrogels, and pH-sensitive hydrogels (Shi et al., 2019).

Thermosensitive hydrogels are widely applied in the biomedical field. This material undergoes a sol-gel transition at body temperature. The thermosensitive modification of chitosan is achieved by adding substances such as β-glycerophosphate, HPMC, and poloxamer to chitosan hydrogels (Blacklow et al., 2019).

β-glycerophosphate is a common thermosensitive material that can thermally induce the migration of protons from chitosan to glycerophosphate, decreasing electrostatic repulsion and promoting the formation of hydrogen bonds among chitosan chains, causing sol-gel transition. Nguyen et al. (2018) integrated chitosan with different concentrations (0.2, 0.4, 0.6, and 0.8%, w/v) of oxidized cellulose nanofibers and prepared thermosensitive injectable hydrogel by sol-gel transition with β-glycerophosphate at body temperature. The hydrogel showed good cytocompatibility with both the MC3T3 pre-osteoblast and L929 fibroblast cell lines. In addition, the hydrogel showed the ability of anti-inflammatory or wound healing (M2) macrophage at 14 days after implantation.

Hydroxypropyl methylcellulose possesses the properties of thermal gels. The hydrophilic groups of HPMC molecules form hydrogen bonds with water molecules at low temperatures, creating cage-like structures that enwrap water molecules. As the temperature rises, the hydrogen bonds break, and water molecules are released from the cage-like structures. The hydrophobic methoxyl groups on the molecular chains of HPMC are exposed and aggregated, eventually forming a 3D network at around 60°C. At this point, the material is in the form of a gel. Wang et al. (2016) prepared a novel thermosensitive hydrogel by mixing chitosan, HPMC, and glycerin. To form a gel network at temperatures lower than 60°C, a high concentration of glycerin was added to break the water sheath of the polymer and promote the formation of hydrophobic areas, lowering the phase transition temperature to body temperature. The results showed that the thermosensitive hydrogel possessed good fluidity, thermosensitivity, low cytotoxicity, and biodegradability, with a pH value of 6.8–6.9 and a gelatinization time of 15 min at 37°C.

Poloxamer is a triblock copolymer consisting of hydrophilic polyoxyethylene at each end and hydrophobic polyoxypropylene in the middle. At the critical micelle temperature, poloxamer molecules form spherical micelles with hydrophobic polyoxypropylene as the core and hydrophilic polyoxyethylene as the shell. As the temperature rises, the accumulation and entanglement of micelles enhance gel formation. For example, by the addition of poloxamer, hUCMSC-exos combined with poloxamer 407 hydrogel existed as a liquid at low temperature and transformed to a semi-solid gel at high temperature, which could fit into the complex and irregular space of diabetic foot wounds. In addition, poloxamer 407 retained and sustainedly released hUCMSC-exos directly onto the injured tissues, which could attract fibroblasts and endothelial cells to promote wound repair (Yang et al., 2020).

Thermosensitive hydroxybutyl chitosan is a kind of hydrogellic chitosan derivative widely applied in the biomedical and pharmaceutical fields. No organic crosslinking agent is needed for the sol-gel transition of this biocompatible and reversibly thermo-responsive hydrogel. This hydrogel can be mechanically reinforced by incorporating rod-shaped chitin through adjusting the network structure (Sun et al., 2018).

pH-sensitive hydrogels are a type of hydrogel whose dimensions vary with ambient pH value. During the wound healing progress, the pH in the wounded area is dynamic. The pH value of normal skin is usually below 5 (Lambers et al., 2006). Once the skin surface is damaged, the underlying tissue with a pH value of 7.4 will be exposed. Chitosan has an approximate pKa of 6.5, making it responsive to constantly changing ambient pH. In the acidic environment during the early stage of wound healing, the expansion of chitosan hydrogels can accelerate cell infiltration and proliferation and facilitate oxygen osmosis. Based on pH variation during wound healing, a pH-sensitive chitosan methacrylate hydrogel with adjustable mechanical properties and swelling ratio was designed (Zhu and Bratlie, 2018). This smart hydrogel would swell at pH ≤ 5.0 and shrink pH ≥ 7.4. The potential applications of such hydrogel include releasing anti-inflammatory drugs during the initial wound healing phase, which will reduce the extent of inflammation in the inflammation stage and avoid overgrowth in the fibroblast proliferation phase.

**FIGURE 6:**
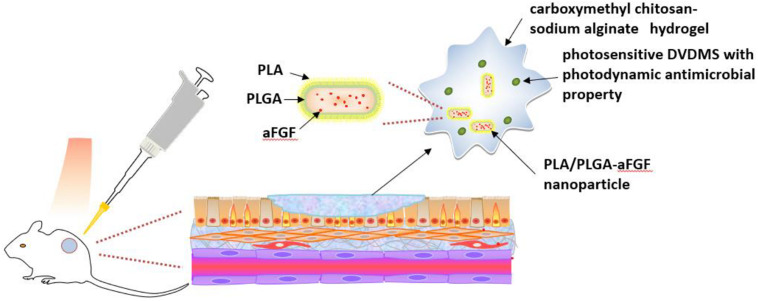
Schematic illustration of light-responsive smart carboxymethyl chitosan-sodium alginate hydrogel which is composed of porphyrin photosensitizer DVDMS and PLGA-encapsulated bFGF nanospheres (Mai et al., 2020).

Light-responsive smart hydrogels can be produced by incorporating photosensitizers into chitosan hydrogels (He et al., 2020). For example, a hybrid hydrogel of carboxymethyl chitosan-sodium alginate containing DVDMS was fabricated (Figure 6). PLA/PLGA nanoparticles embedded with aFGF were added into this hydrogel (Mai et al., 2020). The addition of the DVDMS into carboxymethyl chitosan-sodium alginate hydrogel produced photodynamic antimicrobial properties. Additionally, the hydrogel bulk was helpful for repeatedly photodynamic stimulation, inhibiting bacterial growth while the aFGF content promoted wound healing.

### Self-Healing Chitosan Hydrogels

**FIGURE 7:**
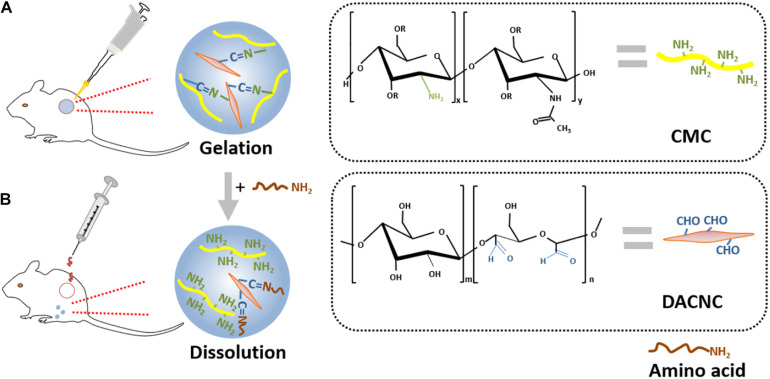
Schematic illustration of a typical example of self-healing hydrogel. **(A)** Hydrogel gelation formed from the Schiff base reaction between aminos of CMC and aldehydes of DACNC. **(B)** Dissolution on-demand. After adding amino acid, new Schiff-base linkages were formed from the aldehyde groups of DACNC and amino groups of amino acid, which leaded to removing of the hydrogel painlessly (Huang et al., 2018).

The Schiff-base bond is a type of dynamic quasi-covalent bond that endows hydrogels with the fluidity of liquids. A self-adapting hydrogel with viscosity, injectability, and self-healing properties was prepared by a dynamic Schiff base reaction between aldehyde groups from oxidized konjac glucomannan and amino groups from protonated chitosan and tranexamic acid (Wang et al., 2020). The hydrogel possessed excellent biocompatibility and antibacterial activity against *S. aureus* and *E. coli*. In addition, this hydrogel was capable of filling irregularly shaped wounds and accelerating the healing process. A self-healing hydrogel was prepared through the Schiff base reaction between the amino groups of carboxymethyl chitosan (CMC) and the aldehyde groups of rigid rod-like DACNC. The cytotoxicity assay and 3D cell culture demonstrated excellent biocompatibility. This hydrogel could be injected into irregular and deep burn wounds, then quickly self-heal to reform if broken during injection, and finally, could be painlessly removed by on-demand dissolution using an amino acid solution (Figure 7) (Huang et al., 2018). Another reversible network structure was acquired by the Schiff base reaction between the aldehyde groups of vanillin and the amino groups of chitosan, followed by hydrogen bonding between the hydroxyl groups of vanillin and the hydroxyl/amino groups of chitosan. This architecture possessed an evenly distributed porous 3D network with a desirable equilibrium between self-healing properties and mechanical strength and, therefore, can be applied to oxygen and nutrient transport in tissue engineering and wound repair (Xu et al., 2018).

### Chitosan/Nanoparticle Hydrogels

Metallic and metallic oxide nanoparticles such as silver, ZnO, copper, gold, and platinum usually exhibit bactericidal activity. Thus, the incorporation of these nanoparticles into chitosan hydrogels will enhance the antibacterial properties of the hydrogels (Jayaramudu et al., 2019). Among these inorganic antibacterial agents, silver ions and compounds have been the most studied. Silver nanoparticles (AgNPs) adsorb onto bacterial surfaces via Coulomb attraction forces, penetrate the cell wall, and then bind to intracellular macromolecules like oxidative metabolic enzymes and DNA, leading to structural variation in the bacterial DNA and impeding bacterial metabolism. Therefore, the incorporation of AgNPs into chitosan hydrogels will enhance their antibacterial properties (Kumar et al., 2020). Jiang et al. (2020) produced hydrogels by adding konjac glucomannan to chitosan, followed by the incorporation of moderate amounts of AgNPs. An *in vivo* tolerance study revealed no skin reactions. Superior antibacterial properties and wound treating ability of the hydrogels were achieved by the sustained release of silver ions. Kumar et al. (2020) verified that sprayable polyvinyl alcohol/chitosan hydrogels with incorporated AgNPs exhibited good antibacterial activities against *S. aureus* and *E. coli.* A positive correlation between antibacterial efficacy and AgNP concentration was seen (Kumar et al., 2020).

ZnO or ZnO-containing compounds, photocatalytic antibacterial agents, kill bacteria by photoinitiation. When exposed to light, these materials produce hydroxyl radicals that damage the structural integrity of the bacterial cell membrane, which is detrimental to bacterial proliferation. Photocatalytic antibacterial materials do not cause secondary pollution and have been widely applied due to their superior bactericidal properties and environmental friendliness. Khorasani et al. (2018) incorporated ZnO nanoparticles into heparinized polyethylene/chitosan hydrogels. The test results showed that the antibacterial efficacy of the as-prepared hydrogels against *S. aureus* and *E. coli* (>70%) was strongly enhanced compared with that of ZnO-free hydrogels (<60%) (Khorasani et al., 2018).

Copper-based antibacterial materials have drawn research attention not only because of their antibacterial properties comparable to those of silver-based materials, but also their cost-effectiveness. For example, chitosan-pluronic hydrogels were produced, followed by the incorporation of CuNPs (Jayaramudu et al., 2020). The hydrogels substantially increased the antibacterial activity against *S. aureus* and *E. coli*, with higher antibacterial efficacy at higher CuNP concentrations.

### Chitosan/Drug Hydrogels

The incorporation of drugs into chitosan-based hydrogels will expand the application scope of the materials, enabling targeted treatments of skin infections, burns, deep wounds, and other skin wounds while the 3D network of the hydrogels can increase the bioavailability of drugs by modulating their release profile.

Microbial infection is the most common factor adversely affecting wound healing, causing swelling, fever, pain, local suppuration, and septicemia. The incorporation of antibiotics into chitosan hydrogels can enhance their efficacy in treating wound infections. For example, mupirocin was added to chitosan-cetyltrimethyl ammonium bromide hydrogels to effectively resist infections caused by *S. aureus* and *Streptococcus pyogenes* (*S. pyogenes*) (Golmohammadi et al., 2020).

Skin burns are a common injury with high morbidity and mortality. The increase in reactive oxygen species (ROS) in cells caused by oxygen and blood deprivation in the early stage of wounds could lead to oxidative stress that is harmful to tissues. Antioxidants such as vitamins A, D, and E accelerate the healing of burned skin. α-Tocopherol, the hydrolyzate of vitamin E, can protect collagen and glycosaminoglycans from oxidative damage. Vitamin D is an antioxidant that alleviates tissue inflammation through regulating antigens involved in differentiation, lymphocyte proliferation, innate immune receptor signal transmission, and chemokine expression. Vitamin A can promote epithelial repair by regulating macrophage functions during the early inflammation stage. Soriano-Ruiz et al. (2020) incorporated vitamin A, D, and E into hydrogel carriers consisting of poloxamer-chitosan-hyaluronic acid to treat skin burns. The *in vivo* tolerance study revealed no skin reactions. The results of partial-thickness burn wound experiments showed that dermal appendages and similar epidermis, dermis, and stratum corneum appeared in animal skins treated with the hydrogel loaded with antioxidant molecules vitamin A, D, and E.

Promoting wound healing for deeply burned skin remains a major challenge. Hesperidin has been reported to possess the capability of repairing deep wounds through inducing VEGF gene expression and stimulating the growth of epithelial cells and collagen deposition (Haddadi et al., 2017). It was loaded into alginate-chitosan hydrogels with various concentrations to enhance deep wound healing. Hydrogels containing 10% hesperidin were not cytotoxic and strongly promoted cell proliferation. The *in vivo* tests verified that wound closure was accelerated compared to treatment with bandages (Bagher et al., 2020).

### Chitosan/Bioactive Substance Hydrogels

The modification of hydrogels with cells and bioactive molecules like growth factors has drawn attention due to its ability to accelerate wound healing by inducing intracellular signaling and stimulating the synthesis of skin repair-related proteins (Guo et al., 2020).

Adipose-derived stem cells (ASCs) can secrete several types of angiogenic growth factors that promote angiogenesis in wounded tissue (Guilak et al., 2006). Cheng et al. (2017) embedded ASCs into thermosensitive chitosan/gelatin hydrogels. *In vitro* tests showed that a higher concentration of VEGF was present in the supernatant of the hydrogel. This hydrogel can be used for therapeutic angiogenesis. BMSCs have been shown to be a candidate for cell therapy for keloids, a kind of benign fibroproliferative tumor in which dermal and subcutaneous extracellular matrices excessively accumulate and outgrow the original wounded area (Fang et al., 2016). A keloid is not only unaesthetic but also prone to becoming cancerous. A previous study reported that Arg-Gly-Asp-grafted hydroxybutyl chitosan hydrogel combined with BMSCs inhibited extracellular matrix synthesis via paracrine signaling, and thus, is a promising candidate for subcutaneous injection treatment for keloids (Qu et al., 2019).

A variety of cytokines participate in repair after skin injury. For example, neutrophils secrete inflammatory cytokines including tumor necrosis factor-α(TNF-α), IL-1β and IL-6 (Burzynski et al., 2019), which play the role of chemotactic inflammatory cells to remove microorganisms. TGF-β secreted by platelets can also promote the recruitment of inflammatory cells during inflammation. Moreover, once the wound area is disinfected. TGF-β can deactivate superoxide production from macrophages which helps to protect the surrounding healthy tissue and prepares the wound for granulation tissue formation (Barrientos et al., 2008). PDGF, and bFGF secreted by platelets and macrophages, respectively, can promote the migration of fibroblasts from the nearby dermis to the wound surface and accelerate the formation of granulation tissue to fill the gap in the wound (Su et al., 2020). In addition, EGF can attract keratinocytes to migrate to the wound site and stimulate the wound to re-epithelialize (Wang et al., 2017). These cytokines all play different roles in the skin repair process. How to incorporate cytokines into the hydrogel to accelerate skin repair has become a research hotspot. Xuan et al. (2020) developed an injectable chitosan hydrogel containing bFGF and silver. The content of bFGF released *in situ* stimulated the proliferation and migration of keratinocytes, endothelial cells, and fibroblasts, promoting collagen formation and re-epithelialization at the wound site. An ethylene glycol chitosan hydrogel containing EGF and bFGF was cured by visible light. EGF stimulated the migration of fibroblasts into the wounded area while bFGF accelerated epithelial regeneration, granulation tissue growth, and collagen formation (Yoo et al., 2018). Compared with pristine ethylene glycol chitosan, the growth factor-loaded hydrogels exhibited better wound healing efficacy.

### Other Chitosan Composite Hydrogels

Different cells play various regulatory roles in the wound healing process. At the inflammation stage, the successively infiltrating neutrophils, macrophages, and lymphocytes clear the invading microorganisms and cell debris and release inflammatory mediators. In the proliferation stage, fibroblasts and endothelial cells promote capillary growth, collagen formation, and granulation tissue development in the wounded area. Keratinocytes proliferate from the edge of the wounds and migrate to the wound areas to promote epidermal formation. However, deficient proliferation and fibroblast, keratinocyte, and endothelial cell migration will delay wound healing. NO can promote the migration and proliferation of cells related to wound healing. *S*-nitroso-*N*-acetyl-DL-penicillamine, an NO donor, was loaded into chitosan/polyvinyl alcohol hydrogels to accelerate angiogenesis and promote healing in chronic wounds. The drug-loaded hydrogel showed a significant increase in cell proliferation and faster recovery of the scratched wound area (Zahid et al., 2019a). The proliferation rate of 3T3 fibroblasts was increased three-fold by NO-releasing chitosan/polyvinyl alcohol hydrogels. *In vitro* cell migration tests showed that the cell migration rate in response to scratches increased by four-fold compared to the control samples.

The application of chitosan hydrogels has been limited by their relatively poor mechanical properties. Therefore, improving the mechanical properties of chitosan hydrogels has become a research pursuit. Lignin-chitosan-polyvinyl alcohol composite hydrogel was shown to possess better mechanical strength than primitive chitosan due to the introduction of lignin into the molecule (Zhang et al., 2019). Lignin is a 3D reticulated biomacromolecule containing active sulfonate and phenolic hydroxyl groups. As a major component of the plant cell wall, lignins can increase the structural strength of plant cells and improve their resistance to an adverse environment. Moreover, the sulfonate groups in lignin can form ionic bonds with the amino groups of chitosan, further increasing the mechanical strength of the composite hydrogels.

In addition, wound dressings with special functions have been developed for diabetic wound treatment. Long-term anoxia is one of the main factors impeding the healing of diabetic wounds (Patil et al., 2019). The pO_2_ of healthy skin ranges between 10 and 40 mmHg, whereas the pO_2_ of the non-wounded skin of diabetic patients is as low as 5 mmHg (Mutluoglu et al., 2013). A previous study reported that a pO_2_ value of 25–100 mmHg was needed for collagen synthesis, angiogenesis, and epithelial regeneration (Mutluoglu et al., 2013). An oxygen-containing biomaterial, MACF chitosan hydrogel, was manufactured and verified to enhance oxygen transportation, thereby promoting collagen synthesis and collagenous fiber rearrangement in diabetic wounds, which are processes beneficial to wound repair (Patil et al., 2019).

## Conclusion and Perspectives

Skin wounds are an extremely common and result from a variety of traumas, such as microbial infections, ulcers, tissue dehydration, and secondary damage. Thus, the development of functional wound dressings capable of promoting wound healing is of great clinical importance. As a kind of native polysaccharide, chitosan is commonly used as wound care material due to its antibacterial and hemostatic properties, as well as the ability to promote granulation tissue growth. Compared to other forms, hydrogels possess the advantages of tissue adhesion and water-retaining properties, and the ability to provide physical barriers for wounds.

In order to improve the effect of chitosan-based hydrogel in accelerating wound healing, researchers have taken many efforts in recent years. For example: (1) Chemical modifications by introducing hydrophilic groups onto chitosan molecules have increased its solubility. Methods such as acylation, carboxylation, quaternization, etherification, and alkylation are often used to introduce aliphatic acyl groups, aromatic acyl groups, carboxyalkyl groups, and quaternary ammonium groups into the molecular structure of chitosan. (2) The mechanical properties of chitosan hydrogel have been improved by adding physical/chemical crosslinking agents such as glutaraldehyde, MBA, and genipin-N, and incorporating carbon nanotubes, polyvinyl alcohol, and lignin to form an interpenetrating network hydrogel. (3) Functional substances such as β-glycerophosphate, HPMC, poloxamer, and photosensitizers have been added to chitosan hydrogels to produce thermosensitive, pH-sensitive, or photosensitive smart hydrogels. This type of hydrogel is widely used in drug-release control, tissue engineering scaffold materials, and medical excipients, and is currently an active research topic. (4) With the addition of substances such as konjac glucomannan and vanillin, the liquid-like fluidity of self-healing hydrogels was achieved through the Schiff base reaction to treat irregularly shaped wounds. This type of hydrogel can self-heal to reform a complete shape after a rupture to provide continuous and effective treatment of wounds. (5) Metal nanoparticles like Ag NPs, ZnO NPs, and Cu NPs have been incorporated into hydrogels to enhance the antibacterial properties. A number of studies verified that the hydrogels containing these inorganic nanoparticles displayed excellent antibacterial effects against Gram-negative *E. coli* and Gram-positive *S. aureus* bacteria. (6) Drugs with antibacterial, antioxidative, and re-epithelializing properties have been incorporated to expand the application of chitosan-containing hydrogels. The 3D network of chitosan hydrogel can accomplish storage promote the proliferation and migration of keratinocytes, endothelial cells, and fibroe and fixation of the drug. In addition, alterations in the degree of swelling will polish drug release, thereby reducing the number of administrations (7) Chitosan hydrogels have been combined with stem cells or/and bioactive substances to stimulate angiogenesis and blasts. Whilst, the hydrophilic network of chitosan hydrogels provide an adaptive environment for cells, improving their survival rate during transplantation.

Nevertheless, there are still some problems for chitosan-based hydrogels to be solved. For example, the poor solubility of chitosan and poor mechanical properties of hydrogels limit their applications in medical devices. Components like drugs, nanoparticles and other substances that were incorporated into the hydrogels make them possess the antibacterial functions. However, certain cytotoxic issues are produced at the same time. How to improve the solubility for chitosan materials to be easily performed or shaped, to minimize the toxicity and to enhance the all advantages while maintaining their intrinsic natures are to be necessary for further investigation. We look forward to the development of multifunctional chitosan hydrogels with promising prospects in wound treatment.

## Author Contributions

PF and YL wrote the manuscript. CK, HQ, and WW corrected and filed up the references. YZ designed and corrected the whole manuscript. RH, LX, and SW proposed the information. All authors contributed to the article and approved the submission.

## Conflict of Interest

SW was employed by the company Ningbo Baoting Biotechnology Co. The remaining authors declare that the research was conducted in the absence of any commercial or financial relationships that could be construed as a potential conflict of interest.

## Abbreviations

3D: three-dimensional
*A. baumannii*: *Acinetobacter baumannii*
ADP: adenosine diphosphate
AF: activated factor
AFGF: alkaline fibroblast growth factor
AgNPs: silver/Ag nanoparticles
ASCs: adipose-derived stem cells
BFGF: basic fibroblast growth factor
BMSCs: bone marrow-derived mesenchymal stem cells
*C. albicans*: *Candida albicans*
CAM: chick embryo chorioallantoic membrane
CCH: chitosan-cetyltrimethylammonium bromide -based hydrogel
CEA: chick embryo angiogenesis
CMCS/CMC: carboxymethyl chitosan
CuNPs: Cu nanoparticles
DACNC: dialdehyde-modified cellulose nanocrystal
DVDMS: photosensitive sinoporphyrin sodium
*E. coli*: *Escherichia coli*
EGF: epidermal growth factor
FTIR: Fourier transform infrared spectroscopy
GCH: glycol chitosanhydrogel
GNB-OM: Gram-negative bacterial outer membrane
GP: glycoprotein
HA: hyaluronic acid
HBC-RGD: hydroxybutyl chitosan-Arg-Gly-Asp
HPMC: hydroxypropyl methylcellulose
HUCMSC-exos: umbilical cord-derived mesenchymal stem cell-derived exosomes
HUVECs: human umbilical vein endothelial cells
IL-1: interleukin-1
IL-6: interleukin-6
IL-8: iInterleukin-8
MACF: methacrylamide fluoride
MBA: methylene bisacrylamide
MDR: multidrug-resistant
MMRSA: *Mupirocin-methicillin-resistant Staphylococcus aureus*
NO: nitric oxide
NPs: nanoparticles
OCNPs: oleoyl-chitosan nanoparticles
OKGM: oxidized konjac glucomannan
*P. aeruginosa*: *Pseudomonas aeruginosa*
PDGF: platelet-derived growth factor
PF3: platelet factor 3
PLA/PLGA: poly(lactic acid)/poly(glycolic acid)
PLGA-bFGF: poly(lactic-co-glycolic acid)-encapsulated basic fibroblast growth factor
PO_2_: oxygen partial pressure
PUFAs: polyunsaturated fatty acids
PVA: polyvinyl alcohol
ROS: reactive oxygen species
*S. aureus*: *Staphylococcus aureus*
*S. cerevisiae*: *Saccharomyces cerevisiae*
*S. pyogenes*: *Streptococcus pyogenes*
*S. typhimurium*: *Salmonella typhimurium*
SA: sodium alginate
SEM: scanning electron microscopy
SeNPs: selenium nanoparticles
SNAP: *S*-nitroso-*N*-acetyl-DL-penicillamine
TGF-β: transforming growth factor-β
TNF-α: tumor necrosis factor-α
TOCNF: tempo-oxidized cellulose nanofiber
TXA2: thromboxane A2
VEGF: vascular endothelial growth factor
VWF: Von Willebrand factor
ZnO: zinc oxide.

## References

[B1] Abd El-Hack M. E. El-Saadony M. T. Shafi M. E. Zabermawi N. M. Arif M. Batiha G. E. (2020). Antimicrobial and antioxidant properties of chitosan and its derivatives and their applications: a review. *Int. J. Biol. Macromol.* 164 2726–2744. 10.1016/j.ijbiomac.2020.08.153 32841671

[B2] Bagher Z. Ehterami A. Safdel M. H. Khastar H. Semiari H. Asefnejad A. (2020). Wound healing with alginate/chitosan hydrogel containing hesperidin in rat model. *J. Drug Deliv. Sci. Technol.* 55:101379. 10.1016/j.jddst.2019.101379

[B3] Barrientos S. Stojadinovic O. Golinko M. S. Brem H. Tomic-Canic M. (2008). Perspective article: growth factors and cytokines in wound healing. *Wound Repair Regenerat.* 16 585–601. 10.1111/j.1524-475X.2008.00410.x 19128254

[B4] Bernkop-Schnürch A. (2018). Strategies to overcome the polycation dilemma in drug delivery. *Adv. Drug Deliv. Rev.* 13 62–72. 10.1016/j.addr.2018.07.017 30059702

[B5] Blacklow S. O. Li J. Freedman B. R. Zeidi M. Chen C. Mooney D. J. (2019). Bioinspired mechanically active adhesive dressings to accelerate wound closure. *Sci. Adv.* 5:eaaw3963. 10.1126/sciadv.aaw3963 31355332 PMC6656537

[B6] Burzynski L. C. Humphry M. Pyrillou K. Wiggins K. A. Chan J. N. E. Figg N. (2019). The coagulation and immune systems are directly linked through the activation of interleukin-1α by thrombin. *Immunity* 50 1033–1042.e6. 10.1016/j.immuni.2019.03.003 30926232 PMC6476404

[B7] Cheng N.-C. Lin W.-J. Ling T.-Y. Young T.-H. (2017). Sustained release of adipose-derived stem cells by thermosensitive chitosan/gelatin hydrogel for therapeutic angiogenesis. *Acta Biomater.* 51 258–267. 10.1016/j.actbio.2017.01.060 28131942

[B8] Clifton L. A. Skoda M. W. A. Le Brun A. P. Ciesielski F. Kuzmenko I. Holt S. A. (2015). Effect of divalent cation removal on the structure of gram-negative bacterial outer membrane models. *Langmuir* 31 404–412. 10.1021/la504407v 25489959 PMC4295546

[B9] Fang F. Huang R.-L. Zheng Y. Liu M. Huo R. (2016). Bone marrow derived mesenchymal stem cells inhibit the proliferative and profibrotic phenotype of hypertrophic scar fibroblasts and keloid fibroblasts through paracrine signaling. *J. Dermatol. Sci.* 83 95–105. 10.1016/j.jdermsci.2016.03.003 27211019

[B10] Farhadihosseinabadi B. Zarebkohan A. Eftekhary M. Heiat M. Moosazadeh Moghaddam M. Gholipourmalekabadi M. (2019). Crosstalk between chitosan and cell signaling pathways. *Cell. Mol. Life Sci.* 76 2697–2718. 10.1007/s00018-019-03107-3 31030227 PMC11105701

[B11] Fukasawa M. Abe H. Masaoka T. Orita H. Horikawa H. Campeau J. D. (1992). The hemostatic effect of deacetylated chitin membrane on peritoneal injury in rabbit model. *Surg. Today* 22 333–338. 10.1007/BF00308742 1392345

[B12] Galván Márquez I. Akuaku J. Cruz I. Cheetham J. Golshani A. Smith M. L. (2013). Disruption of protein synthesis as antifungal mode of action by chitosan. *Int. J. Food Microbiol.* 164 108–112. 10.1016/j.ijfoodmicro.2013.03.025 23624539

[B13] Golmohammadi R. Najar-Peerayeh S. Tohidi Moghadam T. Hosseini S. M. J. (2020). Synergistic antibacterial activity and wound healing properties of selenium-chitosan-mupirocin nanohybrid system: an in vivo study on rat diabetic staphylococcus aureus wound infection model. *Sci. Rep.* 10:2854. 10.1038/s41598-020-59510-5 32071320 PMC7028993

[B14] Guilak F. Lott K. E. Awad H. A. Cao Q. Hicok K. C. Fermor B. (2006). Clonal analysis of the differentiation potential of human adipose-derived adult stem cells. *J. Cell. Physiol.* 206 229–237. 10.1002/jcp.20463 16021633

[B15] Guo Z. Richardson J. J. Kong B. Liang K. (2020). Nanobiohybrids: materials approaches for bioaugmentation. *Sci. Adv.* 6:eaaz0330. 10.1126/sciadv.aaz0330 32206719 PMC7080450

[B16] Haddadi G. Abbaszadeh A. Mosleh-Shirazi M. Okhovat M. A. Salajeghe A. Ghorbani Z. (2017). Evaluation of the effect of hesperidin on vascular endothelial growth factor gene expression in rat skin animal models following cobalt-60 gamma irradiation. *J. Cancer Res. Therapeut.* 14 S1098–S1104. 10.4103/0973-1482.202892 30539852

[B17] He J. Shi M. Liang Y. Guo B. (2020). Conductive adhesive self-healing nanocomposite hydrogel wound dressing for photothermal therapy of infected full-thickness skin wounds. *Chem. Eng. J.* 394:124888. 10.1016/j.cej.2020.124888

[B18] He Q. Gong K. Ao Q. Ma T. Yan Y. Gong Y. (2013). Positive charge of chitosan retards blood coagulation on chitosan films. *J. Biomater. Appl.* 27 1032–1045. 10.1177/0885328211432487 22207609

[B19] Helander I. M. Nurmiaho-Lassila E. L. Ahvenainen R. Rhoades J. Roller S. (2001). Chitosan disrupts the barrier properties of the outer membrane of Gram-negative bacteria. *Int. J. Food Microbiol.* 71 235–244. 10.1016/S0168-1605(01)00609-2 11789941

[B20] Howling G. I. Dettmar P. W. Goddard P. A. Hampson F. C. Dornish M. Wood E. J. (2001). The effect of chitin and chitosan on the proliferation of human skin fibroblasts and keratinocytes in vitro. *Biomaterials* 22 2959–2966. 10.1016/S0142-9612(01)00042-4 11575470

[B21] Huang W. Wang Y. Huang Z. Wang X. Chen L. Zhang Y. (2018). On-Demand dissolvable self-healing hydrogel based on carboxymethyl chitosan and cellulose nanocrystal for deep partial thickness burn wound healing. *ACS Appl. Mater. Interfaces* 10 41076–41088. 10.1021/acsami.8b14526 30398062

[B22] Jackson S. P. Schoenwaelder S. M. Goncalves I. Nesbitt W. S. Yap C. L. Wright C. E. (2005). PI 3-kinase p110β: a new target for antithrombotic therapy. *Nat. Med.* 11 507–514. 10.1038/nm1232 15834429

[B23] Jayaramudu T. Varaprasad K. Pyarasani R. D. Reddy K. K. Kumar K. D. Akbari-Fakhrabadi A. (2019). Chitosan capped copper oxide/copper nanoparticles encapsulated microbial resistant nanocomposite films. *Int. J. Biol. Macromol.* 128 499–508. 10.1016/j.ijbiomac.2019.01.145 30699337

[B24] Jayaramudu T. Varaprasad K. Reddy K. K. Pyarasani R. D. Akbari-Fakhrabadi A. Amalraj J. (2020). Chitosan-pluronic based Cu nanocomposite hydrogels for prototype antimicrobial applications. *Int. J. Biol. Macromol.* 143 825–832. 10.1016/j.ijbiomac.2019.09.143 31715225

[B25] Jiang Y. Huang J. Wu X. Ren Y. Li Z. Ren J. (2020). Controlled release of silver ions from AgNPs using a hydrogel based on konjac glucomannan and chitosan for infected wounds. *Int. J. Biol. Macromol.* 149 148–157. 10.1016/j.ijbiomac.2020.01.221 31982523

[B26] Kassem A. Ayoub G. M. Malaeb L. (2019). Antibacterial activity of chitosan nano-composites and carbon nanotubes: a review. *Sci. Total Environ.* 668 566–576. 10.1016/j.scitotenv.2019.02.446 30856567

[B27] Khan M. Mujahid M. (2018). A review on recent advances in chitosan based composite for hemostatic dressings. *Int. J. Biol. Macromol.* 124 138–147. 10.1016/j.ijbiomac.2018.11.045 30447365

[B28] Khan M. A. Mujahid M. (2019). A review on recent advances in chitosan based composite for hemostatic dressings. *Int. J. Biol. Macromol.* 124 138–147. 30447365 10.1016/j.ijbiomac.2018.11.045

[B29] Khorasani M. T. Joorabloo A. Moghaddam A. Shamsi H. MansooriMoghadam Z. (2018). Incorporation of ZnO nanoparticles into heparinised polyvinyl alcohol/chitosan hydrogels for wound dressing application. *Int. J. Biol. Macromol.* 114 1203–1215. 10.1016/j.ijbiomac.2018.04.010 29634965

[B30] Kumar A. Behl T. Chadha S. (2020). Synthesis of physically crosslinked PVA/Chitosan loaded silver nanoparticles hydrogels with tunable mechanical properties and antibacterial effects. *Int. J. Biol. Macromol.* 149 1262–1274. 10.1016/j.ijbiomac.2020.02.048 32044364

[B31] Kumar A. Kaur H. (2020). Sprayed in-situ synthesis of polyvinyl alcohol/chitosan loaded silver nanocomposite hydrogel for improved antibacterial effects. *Int. J. Biol. Macromol.* 145 950–964. 10.1016/j.ijbiomac.2019.09.186 31669274

[B32] Lambers H. Piessens S. Bloem A. Pronk H. Finkel P. (2006). Natural skin surface pH is on average below 5, which is beneficial for its resident flora. *Int. J. Cosmet. Sci.* 28 359–370. 10.1111/j.1467-2494.2006.00344.x 18489300

[B33] Lee M. Cui Z.-K. Kim S. Baljon J. Wu B. Aghaloo T. (2019). Microporous methacrylated glycol chitosan-montmorillonite nanocomposite hydrogel for bone tissue engineering. *Nat. Commun.* 10:3523. 10.1038/s41467-019-11511-3 31388014 PMC6684526

[B34] Leonhardt E. Kang N. Hamad M. Wooley K. Elsabahy M. (2019). Absorbable hemostatic hydrogels comprising composites of sacrificial templates and honeycomb-like nanofibrous mats of chitosan. *Nat. Commun.* 10:2307. 10.1038/s41467-019-10290-1 31127114 PMC6534699

[B35] Li M. Liang Y. He J. Zhang H. Guo B. (2020). Two-Pronged strategy of biomechanically active and biochemically multifunctional hydrogel wound dressing to accelerate wound closure and wound healing. *Chem. Mater.* 32 9937–9953. 10.1021/acs.chemmater.0c02823

[B36] Li X. Bai H. Yang Y. Yoon J. Wang S. Zhang X. (2019). Supramolecular antibacterial materials for combatting antibiotic resistance. *Adv. Mater.* 31:e1805092. 10.1002/adma.201805092 30536445

[B37] Li Z. Yang F. Yang R. (2015). Synthesis and characterization of chitosan derivatives with dual-antibacterial functional groups. *Int. J. Biol. Macromol.* 75 378–387. 10.1016/j.ijbiomac.2015.01.056 25666853

[B38] Lord M. S. Cheng B. McCarthy S. J. Jung M. Whitelock J. M. (2011). The modulation of platelet adhesion and activation by chitosan through plasma and extracellular matrix proteins. *Biomaterials* 32 6655–6662. 10.1016/j.biomaterials.2011.05.062 21676458

[B39] Mai B. Jia M. Liu S. Sheng Z. Li M. Gao Y. (2020). Smart hydrogel-based DVDMS/bFGF nanohybrids for antibacterial phototherapy with multiple damaging sites and accelerated wound healing. *ACS Appl. Mater. Interfaces.* 12 10156–10169. 10.1021/acsami.0c00298 32027477

[B40] Mohan K. Ganesan A. R. Muralisankar T. Jayakumar R. Sathishkumar P. Uthayakumar V. (2020). Recent insights into the extraction, characterization, and bioactivities of chitin and chitosan from insects. *Trends Food Sci. Technol.* 105 17–42. 10.1016/j.tifs.2020.08.016 32901176 PMC7471941

[B41] Mutluoglu M. Cakkalkurt A. Uzun G. Aktas S. (2013). Topical oxygen for chronic wounds: a PRO/CON debate. *J. Am. College Clin. Wound Special.* 5 61–65. 10.1016/j.jccw.2014.12.003 26199891 PMC4495745

[B42] Nguyen T. H. M. Abueva C. Ho H. V. Lee S.-Y. Lee B.-T. (2018). In vitro and in vivo acute response towards injectable thermosensitive chitosan/TEMPO-oxidized cellulose nanofiber hydrogel. *Carbohydrate Polymers* 180 246–255. 10.1016/j.carbpol.2017.10.032 29103503

[B43] Ong S.-Y. Wu J. Moochhala S. M. Tan M.-H. Lu J. (2008). Development of a chitosan-based wound dressing with improved hemostatic and antimicrobial properties. *Biomaterials* 29 4323–4332. 10.1016/j.biomaterials.2008.07.034 18708251

[B44] Patil P. S. Fathollahipour S. Inmann A. Pant A. Amini R. Shriver L. P. (2019). Fluorinated methacrylamide chitosan hydrogel dressings improve regenerated wound tissue quality in diabetic wound healing. *Adv. Wound Care(New Rochelle).* 8 374–385. 10.1089/wound.2018.0887 31346492 PMC6657299

[B45] Qu C. Bao Z. Zhang X. Wang Z. Ren J. Zhou Z. (2019). A thermosensitive RGD-modified hydroxybutyl chitosan hydrogel as a 3D scaffold for BMSCs culture on keloid treatment. *Int. J. Biol. Macromol.* 125 78–86. 10.1016/j.ijbiomac.2018.12.058 30529347

[B46] Qu J. Zhao X. Liang Y. Zhang T. Ma P. X. Guo B. (2018). Antibacterial adhesive injectable hydrogels with rapid self-healing, extensibility and compressibility as wound dressing for joints skin wound healing. *Biomaterials* 183 185–199. 10.1016/j.biomaterials.2018.08.044 30172244

[B47] Shi Q. Liu H. Tang D. Li Y. Li X. Xu F. (2019). Bioactuators based on stimulus-responsive hydrogels and their emerging biomedical applications. *NPG Asia Mater.* 11:64. 10.1038/s41427-019-0165-3

[B48] Soriano-Ruiz J. L. Calpena-Campmany A. C. Silva-Abreu M. Halbout-Bellowa L. Bozal-de Febrer N. Rodríguez-Lagunas M. J. (2020). Design and evaluation of a multifunctional thermosensitive poloxamer-chitosan-hyaluronic acid gel for the treatment of skin burns. *Int. J. Biol. Macromol.* 142 412–422. 10.1016/j.ijbiomac.2019.09.113 31593719

[B49] Su J. Morgani S. M. David C. J. Wang Q. Er E. E. Huang Y. H. (2020). TGF-β orchestrates fibrogenic and developmental EMTs via the RAS effector RREB1. *Nature* 577 566–571. 10.1038/s41586-019-1897-5 31915377 PMC7450666

[B50] Sun G. Zhang X. Bao Z. Lang X. Zhou Z. Li Y. (2018). Reinforcement of thermoplastic chitosan hydrogel using chitin whiskers optimized with response surface methodology. *Carbohydr. Polym.* 189 280–288. 10.1016/j.carbpol.2018.01.083 29580410

[B51] Verlee A. Mincke S. Stevens C. V. (2017). Recent developments in antibacterial and antifungal chitosan and its derivatives. *Carbohydr. Polym.* 164 268–283. 10.1016/j.carbpol.2017.02.001 28325326

[B52] Wang Q. Z. Chen X. G. Li Z. X. Wang S. Liu C. S. Meng X. H. (2008). Preparation and blood coagulation evaluation of chitosan microspheres. *J. Mater. Sci. Mater. Med.* 19 1371–1377. 10.1007/s10856-007-3243-y 17914628

[B53] Wang T. Chen L. Shen T. Wu D. (2016). Preparation and properties of a novel thermo-sensitive hydrogel based on chitosan/hydroxypropyl methylcellulose/glycerol. *Int. J. Biol. Macromol.* 93 775–782. 10.1016/j.ijbiomac.2016.09.038 27640090

[B54] Wang Y. Xie R. Li Q. Dai F. Lan G. Shang S. (2020). A self-adapting hydrogel based on chitosan/oxidized konjac glucomannan/AgNPs for repairing irregular wounds. *Biomater. Sci.* 8 1910–1922. 10.1039/C9BM01635J 32026892

[B55] Wang Y.-H. Liu C.-C. Cherng J.-H. Fan G.-Y. Wang Y. Chang S.-J. (2019). Evaluation of chitosan-based dressings in a swine model of artery-injury-related shock. *Sci. Rep.* 9:14608. 10.1038/s41598-019-51208-7 31601964 PMC6787046

[B56] Wang Z. Wang Z. Lu W. W. Zhen W. Yang D. Peng S. (2017). Novel biomaterial strategies for controlled growth factor delivery for biomedical applications. *NPG Asia Mater.* 9:e435. 10.1038/am.2017.171

[B57] Xing K. Chen X. G. Kong M. Liu C. S. Cha D. S. Park H. J. (2009a). Effect of oleoyl-chitosan nanoparticles as a novel antibacterial dispersion system on viability, membrane permeability and cell morphology of *Escherichia coli* and *Staphylococcus aureus*. *Carbohydr. Polym.* 76 17–22. 10.1016/j.carbpol.2008.09.016

[B58] Xing K. Chen X. G. Liu C. S. Cha D. S. Park H. J. (2009b). Oleoyl-chitosan nanoparticles inhibits *Escherichia coli* and *Staphylococcus aureus* by damaging the cell membrane and putative binding to extracellular or intracellular targets. *Int. J. Food Microbiol.* 132 127–133. 10.1016/j.ijfoodmicro.2009.04.013 19439383

[B59] Xu C. Zhan W. Tang X. Mo F. Fu L. Lin B. (2018). Self-healing chitosan/vanillin hydrogels based on Schiff-base bond/hydrogen bond hybrid linkages. *Polym. Test.* 66 155–163. 10.1016/j.polymertesting.2018.01.016

[B60] Xuan X. Zhou Y. Chen A. Zheng S. An Y. He H. (2020). Silver crosslinked injectable bFGF-eluting supramolecular hydrogels speed up infected wound healing. *J. Mater. Chem. B.* 8 1359–1370. 10.1039/C9TB02331C 31840731

[B61] Yang J. Chen Z. Pan D. Li H. Shen J. (2020). Umbilical cord-derived mesenchymal stem cell-derived exosomes combined pluronic F127 hydrogel promote chronic diabetic wound healing and complete skin regeneration. *Int. J. Nanomed.* 15 5911–5926. 10.2147/IJN.S249129 32848396 PMC7429232

[B62] Yoo Y. Hyun H. Yoon S.-J. Kim S. Y. Lee D.-W. Um S. (2018). Visible light-cured glycol chitosan hydrogel dressing containing endothelial growth factor and basic fibroblast growth factor accelerates wound healing in vivo. *J. Ind. Eng. Chem.* 67 365–372. 10.1016/j.jiec.2018.07.009

[B63] Zahid A. A. Ahmed R. Raza, Ur Rehman S. Augustine R. Tariq M. (2019a). Nitric oxide releasing chitosan-poly (vinyl alcohol) hydrogel promotes angiogenesis in chick embryo model. *Int. J. Biol. Macromol.* 136 901–910. 10.1016/j.ijbiomac.2019.06.136 31229545

[B64] Zahid A. A. Ahmed R. Ur Rehman S. R. Augustine R. Hasan A. (2019b). Reactive nitrogen species releasing hydrogel for enhanced wound healing. *Annu. Int. Conf. IEEE Eng. Med. Biol. Soc.* 2019 3939–3942. 10.1109/embc.2019.8856469 31946734

[B65] Zhang B. He J. Shi M. Liang Y. Guo B. (2020). Injectable self-healing supramolecular hydrogels with conductivity and photo-thermal antibacterial activity to enhance complete skin regeneration. *Chem. Eng. J.* 400:125994.

[B66] Zhang Y. Jiang M. Zhang Y. Cao Q. Wang X. Han Y. (2019). Novel lignin–chitosan–PVA composite hydrogel for wound dressing. *Mater. Sci. Eng. C.* 104:110002. 10.1016/j.msec.2019.110002 31499949

[B67] Zhu L. Bratlie K. M. (2018). pH sensitive methacrylated chitosan hydrogels with tunable physical and chemical properties. *Biochem. Eng. J.* 132 38–46. 10.1016/j.bej.2017.12.012

